# Mitigation of tau propagation by ultrasound blood‐brain barrier opening

**DOI:** 10.1002/alz70859_098990

**Published:** 2025-12-25

**Authors:** Benoit Delatour, Susana Boluda, Alexandre Carpentier, Amandine Géraudie

**Affiliations:** ^1^ Paris Brain Institute (ICM), Inserm, CNRS, Sorbonne University, Paris, Ile de France France; ^2^ Pitié‐Salpêtrière Hospital, Paris, Ile de France France; ^3^ APHP, Paris, Ile de France France

## Abstract

**Background:**

Evidence from the literature indicates that transient blood‐brain barrier (BBB) opening with ultrasound (aka sonication) can mitigate tauopathies in transgenic mice overexpressing mutations of the MAPT (Microtubule‐Associated Protein Tau) gene. The effects of sonication have not however been assessed on the prion‐like propagation of tau lesions.

**Method:**

P301S mice were stereotactically inoculated with 1) Alzheimer’s brain extracts containing purified tau assemblies to promote an accelerated propagation of lesions, 2) control brain extracts to provide baseline. Half animals in each group were sonicated to transiently and repeatedly open their BBB (1 session/week during 6 weeks). After sacrifice all mice were processed for neuropathological analyses.

**Result:**

Sonication did not significantly modify intrinsic tauopathies in control‐inoculated mice. On the contrary the spreading of tau lesions, in Alzheimer‐inoculated mice, from the initial site of inoculation (hippocampus) to distant brain areas (rhinal cortex, amygdala) was mitigated after repeated sonications. The mechanism of this therapeutic effect is currently examined (microglial phenotyping, biochemical nature of propagated aggregates, modulation of autophagy).

**Conclusion:**

Results indicate that repeated transient BBB opening reduces the accelerated propagation of tau lesions in the brain and suggest therapeutical applications for mitigating disease progression in Alzheimer’s disease and frontotemporal dementia.

